# High parity predicts use of long-acting reversible contraceptives in the extended postpartum period among women in rural Uganda

**DOI:** 10.1186/s40834-018-0059-8

**Published:** 2018-05-09

**Authors:** Ronald Anguzu, Hassard Sempeera, Juliet N. Sekandi

**Affiliations:** 1 Makerere University School of Public Health, Kampala, Uganda; 2 University of Georgia, Global Health Institute, Athens, GA USA

**Keywords:** Long-acting reversible contraception, Postpartum, High parity, Abortion, Uganda

## Abstract

**Background:**

The use of implants and Intra-uterine devices (IUD) during the post-partum period is very low in Uganda especially in rural settings. Long-acting reversible contraceptives (LARC) are known to be the most cost-effective for prevention of unintended pregnancy and unsafe abortions. This study aimed at determining the factors associated with long-acting reversible contraceptive use among women in the extended postpartum period in rural Uganda.

**Methods:**

We conducted a household-based, cross-sectional study among 400 women in two rural communities in Mityana district, central Uganda. Eligible women were aged 15 to 45 years who had childbirth within 12 months of study enrollment in September 2014. The outcome variable was self-reported use of a LARC method, either IUD or implants in the extended postpartum period. The main independent variables were previous childbirths (parity), fertility desire, willingness to use modern contraception, duration of postpartum period and previous pregnancies (gravidity). A logistic regression model was run in STATA v12.0 to compute adjusted odds ratios (AOR) for factors that predicted LARC use statistically significant at *p* < 0.05.

**Results:**

Four hundred respondents had a mean age of 27 years (SD = 12) and only 8.5% reported using a LARC method. Use of IUD and implant was 1.8% and 10.4% respectively. Most women using LARC (44.1%) had five or more childbirths (*p* = 0.01), 70.8% of non-LARC users were willing to use modern contraceptives (*p* = 0.07) and 2.5% ever had an induced abortion. Having five or more childbirths was independently associated with LARC use in the extended postpartum period (AOR = 4.07, 95%CI 1.08–15.4). Willingness to use modern contraception, desire for more children and postpartum duration had no significant association with LARC use in the extended postpartum period.

**Conclusion:**

This study revealed low use of LARC within twelve months of child birth despite women’s willingness to use them. High parity (≥5 childbirths) predicted LARC use. The next logical step is to identify barriers to using LARC in the extended postpartum period and design appropriate interventions to increase access and use especially in multi-parous women.

## Background

The extended post-partum period, defined as the first 12 months after child birth, is known to be a time when there is increased susceptibility to unplanned pregnancy [1, 2]. Mothers in the post-partum period in low and middle income countries (LMIC) are at higher risk of unintended pregnancy than their counterparts in high income countries [3]. This risk of unintended pregnancy is higher among women who are not using effective contraceptives [4, 5]. Failure to use effective contraceptive methods may result in poor child spacing and/or unsafe abortions which further increases the risk of both maternal and child mortality [6, 7]. In the context of HIV prevention, especially in high HIV burdened countries like Uganda, preventing unintended pregnancy also reduces the risk of vertical HIV transmission [8].

Use of these long-acting reversible contraceptives (LARC) provides women with longer periods of protection from pregnancy with less contraceptive failure rates [9]. In Uganda, utilization of modern contraceptives has steadily increased from 8% to 35% over the last decade [10, 11]. However, long-acting reversible contraceptive use has remained as low as 7.8% [12]. This low utilization has been attributed to low awareness, lack or inadequate skilled or trained staff to perform procedures to insert or remove the devices as well as frequent stock outs of family planning methods [9]. One reason for low utilization of modern family planning methods in developing countries is poor access [13–15].

Post-partum utilization of long-acting reversible contraceptives in Uganda remains lower in rural areas despite integration of family planning services into routine postnatal care [11, 16, 17]. Unplanned pregnancies are highly prevalent in Uganda with nearly half of all pregnancies reported to be unplanned especially among women with four or more children and rural dwellers [18]. Our study was motivated by the limited understanding of the facilitators and barriers to LARC use among rural dwelling women in the extended postpartum period. The study therefore aimed at determining the factors associated with use of long-acting reversible contraceptives among rural women in the extended postpartum period in central Uganda.

## Methods

### Study design and setting

This was a household-based, cross-sectional survey conducted in two rural communities in Mityana district, central Uganda between September 2014 and October 2014. Mityana district has three geographical divisions, administratively referred to as Health Sub-Districts (HSDs) or sub-counties [19]. The two HSDs were sites for a recently completed quasi-experimental trial that contained a 12-month postpartum cohort. This cohort were previously recruited and completed participation in a community health intervention aimed at increasing skilled birth delivery through health education of male partners [20]. Mityana district has a total of 58 health facilities; 1 public hospital and 34 health centers levels two, three and four. Two-thirds of all health facilities in Mityana district are government funded and the rest are private [21].

### Study population

Women aged 15 to 45 years were eligible for the study if they resided in the two selected HSDs in Mityana district and had their most recent childbirth, regardless of birth outcome, between September 2013 and September 2014.

### Sample size estimation and selection procedure

A sample of 387 in the extended postpartum period was estimated using the modified Kish Leslie formula (1965). Prevalence (*p)* of 13% for postpartum contraceptive use [22], 5% precision, design effect (DEFF) of 2 due to purposive sampling from two sub-counties and a 90% response rate was used. However, oversampling and screening of 400, i.e. 13 more eligible women was done. From a total of 30 villages with the two study HSDs, we aimed at recruiting at least 13 eligible women from each village. Trained research assistants were guided to household by local Village Health Teams (VHTs) to conduct screening for eligibility and identify study participants. Using an existing list of village residents as the initial sampling frame, phone calls were made to seek initial consent from women and to schedule home visits. Using phone calls, the sampling frame was generated and used to systematically sample eligible respondents. Upon arrival at a potential participant’s home, written informed consent was sought for study participation.

### Data collection

Due to cultural and gender sensitivity, only female research assistants were involved in data collection. Face-to-face interviews were conducted using pre-tested structured questionnaires. Socio-demographic information, reproductive health history like previous childbirths (parity), previous pregnancies (gravidity), contraceptive use and induced abortions was collected. Field level quality control measures were undertaken to ensure consistency, completeness and correctness of collected data.

### Study measurements

#### Dependent variable

The dependent variable was self-reported use of LARC in the extended postpartum period [23]. This was a binary variable measured as user (Yes) and non-user (No). LARC users were self-reported users of implant or intra-uterine devices within this postpartum period. Non-LARC use was self-reported non-use of contraceptives or use of; injectables, oral contraceptive pills (OCP), emergency contraception, male condom, female sterilization, traditional methods (rhythm, withdrawal) and ‘other traditional’ contraception methods at the time of data collection.

#### Independent variables

Main independent variable was previous childbirths (parity) measured as a categorical variable and other covariates included number of previous pregnancies (Gravidity), history of induced (or spontaneous) abortion, willingness to use any family planning or contraceptive, desire for more children and duration of her current post-partum period. Socio-demographic characteristics such as mothers’ age, marital status, religion, level of education and income were collected. Two questions were asked to elicit responses about; (i) willingness to use a contraceptive for prevent pregnancy (yes/no) and (ii) if yes, what their preferred contraceptive was. The first question asked was; “*do you think you will use a contraceptive to delay or avoid pregnancy at any time in the future*”? The second question asked was; *“which contraceptive method would you prefer”*?

### Statistical analysis

Double data entry and cleaning was done in Epi info v7 and then exported to STATA S/E12.0 for statistical analysis. Means and corresponding standard deviations (SD) and percentages were described for all variables and a logistic regression model with robust standard errors used to estimate independent association of explanatory variables and LARC use in the extended post-partum period. At bivariate analysis, variables *p* < 0.15 and biologically plausible variables were included in the multivariable model. Gravidity was excluded from the multivariable model due to collinearity with parity. After adjusting for confounding, the adjusted odds ratios (AOR) and their corresponding 95% confidence intervals were described for independent association with LARC use in the extended post-partum period.

## Results

In this study, a total of 400 women in the extended postpartum period participated (Table 1). The mean age of respondents was 27 years (SD = 12). Among the respondents, 186 (46.5%) were aged 15 to 24 years and most were married (78.5%) and Catholic (49.3%). Majority of respondents were agricultural workers (55.3%) and never had formal education (43.3%). Only 8.5% of respondents were currently using a LARC method, with 1.8% and 10.4% using IUD and implant contraceptives respectively (Table 1).

**Table 1:** Socio-demographic characteristics women in extended postpartum period in Mityana district, 2014, *N* = 400

Characteristic	Frequency	Percentage (%)
Age (years)^a^		
15–24	186	46.5
25–34	161	40.3
35–45	53	13.2
Religion		
Catholic	197	49.3
Protestant	76	19.0
Moslem	85	21.2
Other	42	10.5
Marital status		
Never married	51	12.8
Married/living together	314	78.5
Separated/divorced	35	8.7
Education level		
No school	173	43.3
Primary	151	37.7
Secondary and above	76	19.0
Occupation		
Agricultural work	221	55.3
Salaried employment	30	7.5
Trade/business	66	16.5
Housewife/other	83	20.7
Income (UGX)^b^		
None	74	18.5
1–100,000	273	68.3
> 100,000	53	13.2
LARC use		
No	366	91.5
Yes	34	8.5

^a^Mean age (SD) = 27(12), ^b^1USD = 3370 Ugandan shillings (UGX)

From Table 2, among all respondents, the mean number of pregnancies was approximately 4 (mean = 3.6, SD = 2.3). Two fifths of respondents had 1 to 2 childbirths (45.3%). Three quarters of respondents reported willingness to use any FP method (74.8%) and a modern contraceptive (72.0%) respectively. Three quarters (76.5%) of the respondents expressed desire for more children while half of the respondents (52.9%) were in the first six months of the post-partum period.

**Table 2:** Reproductive health characteristics of women in extended postpartum period in Mityana district, 2014

Variable	Frequency	Percentage (%)
Previous pregnancies (Gravidity)		
1–2	170	42.5
3–4	107	26.8
5+	123	30.7
Previous childbirths (Parity)		
1–2	181	45.3
3–4	123	30.7
5+	96	24.0
Previous spontaneous abortion		
No	332	83.0
Yes	68	17.0
Previous induced abortion		
None	390	97.5
> 1	10	2.5
Willing to use any family planning method		
No	101	25.2
Yes	299	74.8
Willing to use a modern contraceptive		
No	112	28.0
Yes	288	72.0
Desire for more children		
No	94	23.5
Yes	306	76.5
Duration of postpartum period (months)		
< 6	211	52.8
6–9	123	30.9
10–12	65	16.3

In Table 3, nearly twice more women using LARC had at least five childbirths compared to non-LARC users (44.1% vs 22.1%, *p* = 0.01). Approximately twice more women using LARC previously had at least five pregnancies when compared to non-LARC users (50.0% vs 28.0%, *p* = 0.04). Among LARC users and non-users, the proportions of women willing to use MC were comparable (85.3% vs 70.8%, *p* = 0.07) while the proportion of women who desired more children were also comparable (64.7% vs 77.6%, *p* = 0.10). Approximately three quarters (73.8%) of non-LARC users were willing to use any family planning method.

**Table 3:** Bivariate analysis of factors associated with long-acting reversible contraceptive use in Mityana Uganda, 2014

	Non-LARC use, N_1_ (%)		LARC users, N_2_ (%)		
Variable	366 (91.5)		34 (8.5)		*p*-value
	n	%	n	%	
Age^a^					
15–24	174	47.5	12	35.3	0.14
25–34	147	40.2	14	41.2	
35–45	45	12.3	8	23.5	
Religion					
Catholic	180	49.2	17	50.0	0.87
Protestant	69	18.9	7	20.6	
Moslem	77	21.0	8	23.5	
Other	40	10.9	2	5.9	
Marital status					
Never married	48	13.1	3	8.8	0.76
Married/living together	285	77.9	29	85.3	
Separated/divorced	33	9.0	2	5.9	
Education level					
No school	156	42.6	17	50.0	0.55
Primary	138	37.7	13	38.2	
Secondary and above	72	19.7	4	11.8	
Occupation					
Agricultural work	200	54.6	21	61.8	0.28
Salaried employment	27	7.4	3	8.8	
Trade/business	59	16.1	7	20.6	
Housewife/other	80	21.9	3	8.8	
Income^b^					
None	69	18.9	5	14.7	0.89
1–100,000	248	67.8	25	73.5	
> 100,000	49	13.3	4	11.8	
Previous pregnancies (Gravidity)					
1–2	161	43.9	9	26.5	0.04*
3–4	99	27.1	8	23.5	
5+	106	28.0	17	50.0	
Previous childbirths (Parity)					
1–2	172	47.0	9	26.5	0.01*
3–4	113	30.9	10	29.4	
5+	81	22.1	15	44.1	
Previous spontaneous abortion					
No	305	83.4	27	79.4	0.63
Yes	61	16.6	7	20.6	
Willing to use any family planning method					
No	96	26.2	5	14.7	0.15
Yes	270	73.8	29	85.3	
Willing to use modern contraceptive					
No	107	29.2	5	14.7	0.07
Yes	259	70.8	29	85.3	
Desire for more children					
No	82	22.4	12	35.3	0.10
Yes	284	77.6	22	64.7	
Duration of postpartum period (months)					
< 6	198	54.3	13	38.2	0.15
6–9	110	30.1	13	38.3	
10–12	57	15.6	8	23.5	

^a^Mean age (SD) = 27(12), ^b^1USD = 3370 Ugandan shillings, *p<0.05 (statistically significant)

From Table 4, multivariable analysis showed that having five or more childbirths was independently associated with use of LARC in the extended postpartum period (AOR = 4.08, 95%CI 1.08–15.4). Willingness to use MC, desire for more children and duration of postpartum period were not significantly associated with modern contraceptive use in the extended postpartum period.

**Table 4:** Multivariable analysis for predictors of extended postpartum LARC use in Mityana district, 2014

Variable	COR	95% CI	*p*-value	AOR	95% CI	*p*-value
Age^a^						
15–24	1	1		1	1	
25–34	1.38	0.62–3.08	0.43	0.66	0.23–1.92	0.44
35–45	2.58	1.00–6.68	0.05	0.80	0.20–3.29	0.76
Previous childbirths (Parity)						
1–2	1	1		1	1	
3–4	1.45	0.54–3.87	0.46	1.85	0.62–5.57	0.28
5+	2.87	1.23–6.68	0.01*	4.07	1.08–15.4	0.04*
Willing to use modern contraceptives						
Non-MC	1	1		1	1	
MC	2.06	0.78–5.48	0.15	2.23	0.83–6.03	0.11
Duration of postpartum period (months)						
< 6	1	1		1	1	
6–9	1.8	0.81–4.02	0.15	1.67	0.74–3.80	0.22
10–12	2.14	0.85–5.41	0.11	1.94	0.75–5.05	0.17
Desire for more children						
No	1	1		1	1	
Yes	0.53	0.25–1.12	0.09	0.93	0.38–2.27	0.87

^a^Mean age (SD) = 27(12), *p<0.05 (statistically significant)

## Discussion

This study aimed at identifying factors associated with long-acting reversible contraceptive use among women in the extended post-partum period in rural Uganda. We found that only 8.5% of women in the extended postpartum period were using a LARC method. This differed from a survey in urban central Uganda where LARC use among reproductive aged women was nearly three times higher than our study findings [24]. Differences in LARC use between the urban study with higher LARC use and ours could be explained by broad inclusion of contraceptive methods and the differential access to sexual and reproductive health services in rural settings. The urban based survey was health facility-based; therefore, the study population could have been self-selected for female respondents with better healthcare seeking behaviors compared to our household-based sample.

Our findings showed a disparity between willingness to use cost-effective contraceptives and desire for more children suggesting discordance in desired fertility and unmet need for family planning. Few women in our study were using LARCs despite being unwilling to use any family planning method. In addition, most women in our study were willing to use modern contraceptives despite not using any. Possible reasons for the two inconsistencies could be poor physical access, costs and inadequate knowledge of these cost-effective methods. In our study, it is possible that women’s fertility desires reported may reflect their male partner’s fertility desires instead of their own. However, from a study in Rwanda, male spouses to married parous women were supportive their decision to use contraceptives [25]. Studies in rural Uganda and Nigeria revealed decision-making on contraceptive use to be a shared responsibility of a couple [26, 27]. There is a potential need for provision of comprehensive reproductive health services which should include contraceptive counseling.

High parity of women in the postpartum period was strongly associated with using a LARC method and showed a dose-response relationship. As the number of children increased the strength of the likelihood to use LARC also increased. There may be several underlying explanations; parity may indirectly represent an accumulation of contraceptive knowledge through multiple exposures to contraceptive education and/or counseling through each cycle of pregnancy. In addition, these multiparous women may be highly motivated to use LARC because they are more likely to have achieved their desired family size.

Three quarters of non-LARC users in our study reported willingness to use modern contraceptives. Missed opportunities for contraceptives use still exist and highlight the unmet need. Fertility usually returns within the first 6 months after childbirth when not using any contraceptive, we observed that most women in the first 6-month postpartum period were not using any contraceptive method. This unmet need for family planning could be due to poor access to family planning services or low male partner approval for their spouses’ use of contraceptives. Fewer women in their 6 to 12 week postpartum period in Kenya chose IUD (3%) and implant (30%) compared to 1.8% and 10.4% respectively among our respondents [28]. Follow up of both cohorts differed in Kenya and our study at health facility and households respectively. A composite measure of LARC was used in our study compared to independent measures of IUD and implants in the Kenyan study. Continuation rates of LARC have increased due to satisfaction with these methods among women in the postpartum period [29]. There is paucity of evidence from rural Ugandan settings regarding access to and use of LARC among women in the postpartum period. Further research is needed to explore in-depth the contextual barriers to use of LARC in rural communities in Uganda.

Our findings should be interpreted in light of some limitations. This was a cross-sectional study and so causal-temporal relationships could not be ascertained. Reporting bias could have occurred which we attempted to minimize by use of female research assistants who probed these sensitive reproductive health questions such as contraceptive use or abortion. Our study sample size was adequately powered and the study areas were purposively selected to increase our strength of findings and representativeness of this rural community respectively.

Our study findings could have broad application and relevance to similar settings especially in low-resource settings and geographically hard-to-reach populations where access to LARC remains a major challenge. The unmet need for LARC methods in these settings therefore begs for more innovative approaches to overcome the most prevalent barriers: the high client out-of-pocket costs, lack of trained providers and weak supply chains [30, 31]. A recently published study done in Uganda showed that using a combination of family planning vouchers and social franchise scheme significantly increased contraceptive access for the hard-to-reach populations [30].

## Conclusion

This study showed low use of LARC methods within the first twelve months of child birth in rural Uganda. Women having five or more children were more likely to use LARC methods. Number of children predicted LARC use. Most women in early postpartum were not using LARC, this may suggest an unmet need for limiting birth in this rural setting. There is a need to identify barriers to LARC use to inform the design of interventions to increase use of LARC especially among multi-parous women.

## Abbreviations

IUD: Intra-uterine device
LAM: Lactation amenorrhea method
LARC: Long-acting reversible contraception
LMIC: Low and middle-income countries
MNCH: Maternal, newborn and child health
OCP: Oral contraceptive pills
VHT: Village health teams
